# Mr. Atkinson's Case of Monstrosity

**Published:** 1804-07-01

**Authors:** James Atkinson

**Affiliations:** York


### *To the Editors of the Medical and Physical Journal.*

GENTLEMEN,

AN uncommon Case of Monstrosity in the fœtus of a woman having lately occurred, you may possibly think it not altogether unacceptable to your Readers. Inasmuch as it vindicates, in this instance, the veracity of an author who has been rather playful in his ideas upon the subject, the concurring testimony of a fact may be of some advantage.

The woman had twins near the seventh month of pregnancy. She was uncommonly large, and the flow of waters at birth immense.*

The first born child was perfect; the other wanted head, neck, arms, and chest. The ribs, upon dissection, were apparent, though minute, without any regular cavity of thorax.

As a similar case exists in Palfyn, and nothing more extraordinary happened at the time of labor, or previous to it, on that head I shall forbear troubling you with a more minute account.

Palfyn

---

* The fœtus was given to me by my friend here, Mr. Rowntree, who attended the labor.

Palfyn having given a description of the abdomen and pelvis, it induced me also to examine those parts; for it is instructing to observe how the powers of nature enable her to effect varieties, under circumstances and form apparently the same.

Upon the abdomen, above the navel, there was a kind of blind hole, which might have led to the supposition, that it was a passage communicating internally with vessels. Neither outward nor inward examination shewed any thing but an imperfect indentation.

Corresponding with the situation of the shoulders, there was on each side a considerable sac, which gave the feel of a viscus beneath; and from the extent of the cavity on the right side, it particularly appeared to include the liver. It also evidently contained a fluid, which was the cause of the projecting roundness. Upon opening these sacs, not any thing but a meconium-like fluid was contained.

The left cavity was somewhat intersected by small delicate ligamentary webs, interspersed with white filamentous bands, like flattened nerves.

The septum betwixt the two cavities was transparent and ligamentous, surrounded by membrane.

The abdomen, from the smallness of the fœtus, necessarily afforded very little space betwixt the parietes of the cavity and its contents.

The liver was the most conspicuous organ, extending over both sides, in two large distinct lobes, included within a very evident and beautiful sac.

Each larger lobe was distinguished by reticular lines, as in the conglomerate gland, without gall bladder.

From their uniting part arose the umbilical vessels, meeting as usual the urachus.

I could not perceive any stomach, spleen, or pancreas; nor discover any precise direction or mark of colon.

The intestines were very short and round, evidently terminating in the rectum, which was of natural conformation; the ileum was lying on each side the centre of the abdomen.

The funis, on quitting the navel, projected with a knot. After tracing the urachus, and the vessels from the liver to the funis, I opened the small pouch of the funis, which was attached to the navel. In it were contained several short convolutions of the ileum, with an evident caput coli, and an appendicula vermiformis.

This was a congenite umbilical hernia, which had almost eluded observation, having no trace of it before it was opened.

Somewhat

Somewhat lower than the natural situation of the kidneys, though not included cellular substance, were two very small glandular looking bodies of an oblong shape; a small white band or vessel traversed downwards from them towards the pelvis.

It being a female fœtus, I sought for the uterus, &c.

There was not any uterus or bladder, nor the appendages of the uterus, unless the two preceding glands were ovaria, which they did not appear to be.

The external passage of the urethra was pervious to a small wire, being a cul de sac, or was lost as such in the cellular membrane superficially under the skin.

The vagina had the semblance of being closed, yet a probe passed readily; but it passed into the urachus in a continued and obvious way.

There was an abdomen bounded by a membranous diaphragm, without apertures or ligamentous structure.

Should any gentleman of a lively turn be disposed to amuse himself with M. Palfyn's recital of the original cause of the production of this, and of such like formations, he will find it in his Traite des Monstres.

Having myself, little genius for such enquiries, I solicit others to undertake them, with this request, however, that the public be indulged by facts before they exercise their talents upon the physiology. For, sometimes, I am apt to think, there are to be perceived little monstrosities as well of the mind as in the body.

> "Quo vobis mentes, rectæ quæ stare solebant

> Antehac, clementi sese flexere viai?"

I am, &c.

*York, June* 11, 1804. JAMES ATKINSON.

